# The effect of multiple vitrectomies and its indications on intraocular pressure

**DOI:** 10.1186/s12886-019-1187-x

**Published:** 2019-08-08

**Authors:** Hrvoje Kovacic, Roger C. W. Wolfs, Emine Kılıç, Wishal D. Ramdas

**Affiliations:** Department of Ophthalmology, Erasmus Medical Center, PO Box 2040, Rotterdam, CA 3000 The Netherlands

**Keywords:** Intraocular pressure, Glaucoma surgery, Retinal detachment, Vitrectomy

## Abstract

**Background:**

To assess the relationship between different indications for trans pars plana vitrectomies (PPV’s) and the intraocular pressure (IOP), and the effect of multiple PPV’s on the IOP. We also examined whether there were differences in the number of IOP-lowering medications or surgeries before and after PPV.

**Methods:**

A retrospective study including all patients that underwent at least one PPV in the period from 2001 till 2014 at our clinic. Medical records of all patients were reviewed and clinically relevant data were entered in a database. Generalized estimating equations models for repeated measurements were used to examine the effect of the number of PPV’s on the IOP and on the risk of undergoing glaucoma surgery, for each of the indications for PPV.

**Results:**

Of 1072 PPV’s 447 eyes fulfilled the inclusion criteria. The IOP increased with 3.0 mmHg after a PPV with indication retinal detachment (*p* < 0.001), but remained stable after PPV for epiretinal membrane (*p* = 0.555), macular hole (*p* = 0.695), and vitreous hemorrhage (*p* = 0.787). At the end of the follow-up period the number of IOP-lowering medications was significantly higher compared to baseline, except in the macular hole group (*p* = 0.103). Also, the number of eyes that underwent glaucoma surgery was significantly higher compared to the fellow (not-operated) eyes (*p* < 0.001). There was a significant association between the number of PPV’s and the final IOP for the indication retinal detachment (*p* = 0.009), and between the number of PPV’s and glaucoma surgery (odds ratio [95% confidence interval]: 2.60 [1.62–4.15]).

**Conclusions:**

The IOP rises significantly after PPV with indication retinal detachment. This association was not found for other indications for PPV. Also, the risk of IOP-lowering surgeries was higher after PPV, but not different between the PPV indications. The IOP should be monitored carefully after PPV, since there may be a higher risk of secondary glaucoma.

## Background

The introduction of pars plana vitrectomy (PPV) has led to a great advancement in treating conditions of the posterior segment of the eye. [1, 2] Over the years the number of indications for PPV’s has increased including retinal detachment, [3, 4] epiretinal membrane, [5] macular hole, [6] vitreous hemorrhage, [7, 8] floaters, [9] and vitreomacular traction syndrome. [10] Even though PPV has become indispensable in the treatment of many eye diseases, there are still some serious complications that can occur postoperatively. One of them is an elevated intraocular pressure (IOP), which is a major risk factor of glaucoma: an optic neuropathy that causes irreversible progressive visual field loss. Numerous studies have evaluated the postoperative effects of PPV on the IOP. In the early postoperative period an elevation of 5–35% of the IOP has been observed. [11, 12] On the late postoperative effects there have been conflicting results. [13–18] Some studies reported an increase in the IOP or the number of IOP-lowering medications after PPV, [13, 14] with some suggesting that the IOP-increase may be affected by the indication for the PPV. [15] Several other studies could not observe an increase in IOP after vitrectomy. [16–18]

Although many studies have described the influence of PPV on IOP, the influence of the indication and the number of PPV’s on the long-term IOP has not yet been clarified even though repeated PPV’s are not uncommon. The aim of the present study was first to assess the relationship between the most common indications for a PPV and the IOP. Secondly, we examined whether there were differences in the number of IOP-lowering medications or surgeries before and after PPV. Thirdly, we assessed whether the number of PPV’s was associated with a higher IOP or a higher risk of undergoing glaucoma surgery.

## Methods

### Study population

For this retrospective study we selected all patients that underwent at least one PPV in the period from October 2001 till September 2014 at the department of Ophthalmology of the Erasmus University Medical Center, Rotterdam, the Netherlands.

The medical records of all patients were reviewed and clinically relevant data were entered in a database. Data of the last preoperative visit, postoperative follow-up data at 1 day, < 2 weeks, 1–3 months, 1 year, and > 3 years (last visit) were collected. The following pre- and postoperative data were recorded: age, gender, visual acuity, refraction, intraocular lens status, axial length, IOP, and use of ophthalmic medication. Peroperative data consisted of: date of surgery, operated eye, indication, applied tamponade, usage of argon laser, and gauge of vitrectomy device.

The following indications for PPV were included: retinal detachment, epiretinal membrane, macular hole, and vitreous hemorrhage. Exclusion criteria included: eye trauma, intraocular tumor, scleral buckling procedure, endophthalmitis, suprachoroidal hemorrhage, treatment because of previous performed complicated cataract surgery (such as intraocular lens luxation, posterior capsule defect with vitreous loss, or retained lens material), neovascular glaucoma, uveitis with vitritis, and vitreous biopsy.

### IOP measurement, PPV surgery and follow-up

The IOP was measured at each visit using Goldmann applanation tonometry (Haag-Streit, Köniz, Switzerland). The device had been calibrated conform manufacturers standards. The number of IOP-lowering medications was calculated by adding the number of different categories of medication. The categories were: beta-blockers, prostaglandin-analogues, carbonic anhydrase inhibitors, alfa2-agonists, and oral acetazolamide. Fixed combinations of eye drops were calculated as two separate drugs.

The PPV was performed by one of three experienced surgeons. The surgical procedure consisted of a 20, 23 or 25-gauge PPV with or without additional argon laser coagulation. Applied tamponade included: air, gas, or silicone oil. Details on the surgical procedures are described elsewhere. [19]

Follow-up time was measured as the difference between the last preoperative visit for the first PPV till the last visit. If the PPV in the timeframe (from October 2001 till September 2014) was not the first PPV of the patient, we reviewed the medical records again to find out when the patient underwent the first PPV (i.e. before October 2001).

### Statistical analysis

The effect of peroperative variables were assessed using ANOVA-tests, chi-square and, if the number of cases in a subgroup analysis was too low, Fisher’s exact tests. The following groups were made according to PPV indications: retinal detachment, epiretinal membrane, macular hole, and vitreous hemorrhage. To remove the possible effect of differences in baseline characteristics between the different groups on the IOP, we analyzed each PPV indication separately. If a patient used IOP-lowering medication at a visit or had a history of IOP-lowering laser in the included eye(s), we recalculated the IOP of that visit. This was to be able to compare untreated IOP-levels with treated IOP-levels. Based on a reported average of a 30% reduction in IOP caused by IOP lowering medication, reported in a meta-analysis of randomized clinical trials, IOP values of those receiving medication were divided by 0.7 to estimate the untreated IOP. [20, 21] Patients that underwent surgery for glaucoma were excluded from this analysis. A subset comparison was performed to analyze the differences between the operated and non-operated fellow eyes using paired t-tests.

In some cases both eyes of a patient were included. To account for the within-patient inter-eye correlations generalized estimating equations were applied. General linear mixed models for repeated measurements were used to examine the effect of the number of PPV’s on the IOP for each of the PPV indications. An unstructured correlation matrix was applied for the models. These models were adjusted for age, gender, axial length, and follow-up time. No adjustment was done for the lens status, because cataract surgery may be performed before or during the study. Furthermore, previously we did not find a significant difference in IOP if measured with Goldmann applanation tonometry between PPV and PPV combined with cataract surgery. [19] Similar analyses were performed to calculate odds ratios (ORs) with corresponding 95% confidence intervals (CIs) for the effect of the number of PPV’s on the risk of requiring glaucoma surgery. These analyses were adjusted for the above-mentioned variables and were done separately for each of the PPV indications (if possible). All statistical analyses were performed using SPSS v22.0 for Windows (SPSS Inc., Chicago, IL, USA). A *p*-value of < 0.05 was considered statistically significant.

The datasets used and/or analyzed during the current study are available from the corresponding author on reasonable request.

## Results

A total of 1072 PPV’s were performed in 615 patients. Of these, 409 patients (447 eyes) fulfilled the inclusion criteria. Table 1 presents the (preoperative) general characteristics of the study population according to PPV indication. The mean (median; range) follow-up was 2.6 (1.5; 0.1–17.4) years. None of the patients had a history of glaucoma surgery before they underwent PPV. The distribution of type of tamponade for the different PPV indications is presented in Table 2.

**Table 1:** General (preoperative) characteristics of the study eyes according to PPV indication, presented as mean ± s.d. unless stated otherwise

PPV indication	Retinal detachment (*N* = 139)	Epiretinal membrane (*N* = 124)	Macular hole (*N* = 72)	Vitreous hemorrhage (*N* = 112)
Age (years)	69.6 ± 11.0	74.7 ± 7.7	72.5 ± 8.5	70.6 ± 11.8
Women, *N* (%)	51 (36.7)	71 (57.3)	51 (70.8)	56 (50.0)
Best visual acuity	0.3 ± 0.4	0.5 ± 0.2	0.3 ± 0.2	0.2 ± 0.4
Baseline IOP (mmHg)	14.2 ± 4.9	14.5 ± 3.6	15.2 ± 4.7	14.9 ± 6.5
Baseline use of IOP-lowering Rx, *N* (%)	1 (0.7)	0 (0.0)	0 (0.0)	1 (0.9)
Pseudophakic eyes, *N* (%)	33 (23.7)	45 (36.3)	22 (30.6)	51 (45.5)
Axial length (mm)	24.9 ± 1.6	23.9 ± 1.5	23.9 ± 1.9	23.6 ± 1.3
History of diabetic mellitus, *N* (%)	13 (9.4)	22 (17.7)	8 (11.1)	50 (44.6)
History of diabetic retinopathy, *N* (%)	3 (2.2)	4 (3.2)	1 (1.4)	21 (18.8)
History of hypertension, *N* (%)	13 (9.4)	27 (21.8)	15 (20.8)	24 (21.4)

*PPV* pars plana vitrectomy*s.d.* standard deviation, *IOP* intraocular pressure, *Rx* ophthalmic medication

**Table 2:** Peroperative used tamponade of the study eyes according to PPV indication, presented as number (percentage)

PPV indication	Retinal detachment (*N* = 139)	Epiretinal membrane (*N* = 124)	Macular hole (*N* = 72)	Vitreous hemorrhage (*N* = 112)
Tamponade, *N* (%)				
Air	15 (10.8)	100 (80.6)	2 (2.8)	100 (89.3)
SF6-gas	93 (66.9)	23 (18.5)	62 (86.1)	11 (9.8)
Other gas (e.g., C3F8)	10 (7.2)	1 (0.8)	5 (6.9)	0 (0.0)
Silicone oil	21 (15.1)	0 (0.0)	3 (4.2)	1 (0.9)

*PPV* pars plana vitrectomy, *SF6* sulfurhexafluoride, *C3F8* perfluoropropane

Concerning the peroperative variables, no association was observed between the final IOP and the type of tamponade (*p* = 0.752) and usage of argon laser or retinal cryocoagulation (*p* = 0.218). Although patients who underwent PPV using a 20-gauge had a higher final IOP compared to those who underwent a 23 or 25-gauge PPV, this difference was not significant (20.5 mmHg, 15.4 mmHg, and 15.5 mmHg, respectively; *p* = 0.073).

Table 3 displays the differences in the baseline and the final IOP for the operated and non-operated eyes for each PPV indication. The IOP increased with 3.0 mmHg after PPV with indication retinal detachment (*p* < 0.001), but remained stable after PPV for the other indications. In the non-operated eyes no significant increase in baseline and final IOP was found for the different PPV indications. The axial length and IOL-status showed no significant correlation to either baseline IOP or final IOP, in both operated and non-operated eyes. Table 4 shows the number of IOP-lowering medications for the different PPV indications. Of the eyes that underwent PPV for retinal detachment, 14 eyes required IOP-lowering medication using a total of 25 IOP-lowering medications. At the end of the follow-up period the total number IOP-lowering medications was higher than at baseline (*p* < 0.001, *p* = 0.034, *p* = 0.103, and *p* = 0.034 for the PPV indications retinal detachment, epiretinal membrane, macular hole, and vitreous hemorrhage, respectively). A total of 11 patients underwent glaucoma surgery compared to 0 at baseline. Surgery consisted of glaucoma drainage device (*N* = 7 eyes), trabeculectomy (*N* = 2 eyes), surgical peripheral iridectomy (*N* = 1 eye), and one patient underwent trabeculectomy followed by a glaucoma drainage device (*N* = 1 eye). After exclusion of the eyes that underwent glaucoma surgery after PPV, IOP was still significantly higher compared with baseline (*p* < 0.001). During the follow-up period no surgeries for glaucoma were performed in the non-operated fellow eyes.

**Table 3:** Mean IOP of the study eyes according to PPV indication, presented as mean ± s.d. unless stated otherwise

PPV indication		Retinal detachment (*N* = 139)	Epiretinal membrane (*N* = 124)	Macular hole (*N* = 72)	Vitreous hemorrhage (*N* = 112)
Operated eyes					
Preoperative	Baseline IOP (mmHg)	14.2 ± 4.9	14.5 ± 3.6	15.2 ± 4.7	14.9 ± 6.5
Postoperative	Final IOP (mmHg)	17.2 ± 9.4	14.7 ± 4.3	15.5 ± 4.9	15.0 ± 7.0
	p-value	< 0.001	0.555	0.695	0.787
Non-operated fellow eyes^a^					
		(*N* = 96)	(*N* = 94)	(*N* = 52)	(*N* = 69)
Preoperative	Baseline IOP (mmHg)	15.5 ± 5.9	15.6 ± 3.1	15.2 ± 3.1	15.7 ± 4.9
Postoperative	Final IOP (mmHg)	16.1 ± 5.0	15.2 ± 3.7	15.9 ± 5.2	14.5 ± 4.1
	*p*-value	0.092	0.272	0.300	0.023

^a^ patients who underwent PPV on both eyes were excluded, *PPV* pars plana vitrectomy, *s.d.* standard deviation; *IOP* intraocular pressure

**Table 4:** Postoperative characteristics of the study eyes according to PPV indication, presented as mean ± s.d. unless stated otherwise

PPV indication	Retinal detachment (*N* = 139)	Epiretinal membrane (*N* = 124)	Macular hole (*N* = 72)	Vitreous hemorrhage (*N* = 112)
Use of IOP-lowering Rx, *N* eyes (%)	14 (10.1)	5 (4.0)	3 (4.2)	5 (4.5)
Total *N* of IOP-lowering Rx^a^	25	7	4	12
Follow-up, median (IQR; months)	17.3 (3.8–49.5)	16.0 (5.6–36.1)	18.3 (7.4–33.4)	25.8 (5.7–47.9)
Number of PPV	2.6 ± 1.9	1.6 ± 0.9	2.0 ± 1.2	1.9 ± 1.1

*PPV* pars plana vitrectomy, *s.d.* standard deviation, *IOP* intraocular pressure, *Rx* ophthalmic medication, *IQR* interquartile range, ^a^ cumulative number of different categories of IOP lowering Rx: beta-blockers, prostaglandins, carbonanhydrase inhibitor, alfa-agonists, and oral acetazolamide

The average number of PPV’s was 2.8 (range 1–10). The multivariate analyses showed a significant association between the number of PPV’s and the final IOP for the indication retinal detachment (1.11 mmHg decrease in IOP per PPV; *p* = 0.009). Patients who underwent more than five PPV’s for the indication retinal detachment more often suffered from hypotony compared to those who underwent a fewer number of PPV’s. This association was not present for the indications epiretinal membrane (*p* = 0.168), macular hole (*p* = 0.573), and vitreous hemorrhage (*p* = 0.921). Regarding glaucoma surgery, for each performed PPV the risk of undergoing glaucoma surgery increased 2.60 fold (95% CI: 1.62–4.15), especially for the indication retinal detachment (OR: 4.60 [95% CI: 1.54–13.75]). For the other PPV indications the number of glaucoma surgeries was too low to perform subgroup analyses.

## Discussion

The current study shows that among the most common PPV indications patients who underwent PPV because of retinal detachment had a significantly higher IOP postoperatively compared to the preoperative IOP. Compared to the non-operated eyes, glaucoma surgery was performed more often on eyes that had undergone a PPV. Also the risk of undergoing glaucoma surgery increased with the number of PPV’s.

Since elevated IOP plays a crucial role in the development of glaucoma, it is important for clinicians to know the effect of a PPV on the IOP. Patients with or suspect for glaucoma are more prone to develop a higher IOP in the first month postoperative compared to patients not suspect for glaucoma. [22] As mentioned in the Introduction in the early postoperative period, less than 72 h after PPV, an elevation of 5–35% of the IOP has been reported. [11, 12] This rise in IOP is probably due to postoperative inflammation, effects of the steroids, or a reaction to the peroperative tamponade used. Concerning the peroperative variables, on one hand some studies reported an increased IOP after PPV with expanding gas or silicon oil tamponade, [23–29] while on the other hand silicone oil tamponade did not appear to be associated with persisting high IOP or progressive optic disc changes 48 months after PPV. [30] A reason that the present study could not confirm an association might be the size of different subgroups of tamponades. Almost 50% of all tamponades consisted of SF6-gas. Due to this the size of the other subgroups of tamponade were small. Furthermore, the use of silicone oil as tamponade is often not a first choice, but is usually performed after a first PPV fails to be successful. As expected peroperative argon laser or retinal cryocoagulation did not affect the IOP. This finding is in line with another study. [22] Although, one study reported an association between the IOP 1 day after PPV and the number of laser photocoagulations, [31] this effect is likely not to persist on the long-term IOP. Regarding the applied gauge PPV, it has been suggested that a smaller sclerotomy in the pars plana of the eye, i.e. a higher gauge, may result in more hypotony early post-operative after PPV. [32] This is explained by the fact that the sclerotomy in a 23 or higher gauge PPV are not sutured like in a 20-gauge PPV procedure resulting in more wound leakage. The hypotony usually resolves spontaneously once the sclerotomies heal adequately. In the present study the post-operative IOP was found to be higher for 20-gauge PPV compared to 23 or 25-gauge PPV, though not statistically significant.

Regarding the late postoperative period, multiple studies assessed the effect of a PPV on the IOP. Chang found that a PPV could increase the risk of the development of glaucoma in up to 15–20% of the eyes that are operated. He theorized that oxidative stress affects the cells of the trabecular meshwork, causing a rise in IOP. [13] Wu et al. reported a significant increase in the number of vitrectomized eyes (19.2%) with an IOP ≥ 24 mmHg or a rise in IOP ≥ 5 mmHg, compared to non-operated fellow eyes (4.5%). [14] Fujikawa et al. reported a mean increase in IOP of 0.7 mmHg after PPV for macular hole, which was significantly higher than the 0.3 mmHg increase after PPV for epiretinal membrane. [15] On the contrary, Mi et al. found no significant increase in IOP after PPV for epiretinal membrane or macular hole in eyes without pre-existing glaucoma. [16] Yu et al. compared vitrectomized eyes with fellow control eyes in 441 patients and found no significant differences in mean IOP or in the development of glaucoma. [17] Lalezary et al. researched 101 eyes after elective PPV. They found no significant increase in IOP of > 4 mmHg in operated versus non-operated eyes. [18] The present study found a significant increase in IOP of 3.0 mmHg (21.1%) after PPV with indication retinal detachment, however not after PPV for epiretinal membrane (0.2 mmHg), macular hole (0.3 mmHg) and vitreous hemorrhage (0.1 mmHg). This difference might be caused by synechial angle closure (partial or significant), though no gonioscopy was performed to confirm this.

Little is known about the effects of multiple PPV’s on the IOP. We observed a significant association between the number of PPV’s and the final IOP for the indication retinal detachment, though not for the other indications. This is in line with a study on 70 eyes by Tranos et al. [30] They reported a mean of two PPV’s for retinal detachment to be a risk factor for developing progressive optic disc changes. Nevertheless, the significance disappeared when adjusting for the 9% that developed a hypotony. Furthermore, they did not assess other indications for PPV. [30] We found a significant decrease in IOP after multiple PPV’s for retinal detachment. However, after excluding the 6 eyes that developed hypotony (IOP ≤ 4 mmHg), the significant effect disappeared. These were all eyes that underwent multiple PPV’s. Only a few eyes can change this effect on IOP. Thus the IOP rises after PPV for retinal detachment, but probably the effect of the number of PPV’s on the final IOP is not much. The significantly lower IOP may also be explained by the fact that multiple PPV’s result in dysfunction of the ciliary body caused by the removal of proliferative vitreous membranes that cover the ciliary body. Furthermore, it is very difficult to estimate the untreated IOP after glaucoma surgery, and as a consequence, patients who underwent glaucoma surgery were excluded from the analyses in which the final IOP was entered as the outcome. Thus the eyes that developed a hypotony and the exclusion of eyes that underwent glaucoma surgery might explain the present IOP-lowering effect of multiple PPV’s. Inclusion of eyes that underwent glaucoma surgery showed a strong association between the number of PPV’s and the risk of undergoing glaucoma surgery (OR: 2.60 [95% CI: 1.62–4.15]). These results are in line with another recent study (OR 2.35 [95% CI: 1.17–4.70]). [33]

Since treatment of IOP is the keystone in preventing and managing glaucoma, we also looked at IOP-lowering interventions, such as medication and surgery. At baseline only 2 eyes (0.4%) were on IOP-lowering medication, which increased to 27 eyes (6.0%) at the end of follow-up, though not statistically significant (*p* = 0.117). Chang also reported an increase in the number of IOP-lowering medication in vitrectomized eyes compared with the fellow control eyes (1.79 vs. 0.65, respectively). [13] To limit the effect of other attributing factors like time-lag, we did a paired subset comparison with the non-operated eyes. It showed that in the non-operated eyes no glaucoma surgery was performed during follow-up. This observation shows that the effects of PPV are not only limited to a rise in IOP, but also have a clinical impact where glaucoma surgery is needed. Nonetheless, it should be emphasized that treatment of IOP does not necessarily mean that a person suffers from glaucoma, but he/she has an increased risk to develop glaucoma. From an ethical point of view it is not possible to screen someone for glaucoma if the person requires a PPV for retinal detachment or a vitreous hemorrhage. Moreover, monitoring glaucoma using visual field progression has been proven to be a difficult task and visual field defects can also occur after retinal detachment. Therefore, we only focused on IOP and IOP-lowering treatment (either with medication or surgery) instead of a diagnosis of glaucoma. Besides, in many cases follow-up was too short (57.7% had a follow-up < 2 years) to detect glaucomatous damage at the optic nerve or visual field loss (see further).

Due to its retrospective design the current study has a few limitations. First, the follow-up of patients was limited in patients who had no complaints postoperatively. These patients might have developed an unnoticed high IOP. However, the non-inclusion of these patients would have resulted in an underestimation of our findings. Secondly, the probability of finding an increase in IOP is higher in eyes with retinal detachments (compared to the other indications), because they often have a lower pre-operative IOP than healthy eyes. Furthermore, recurrent retinal detachments are not uncommon and may result in multiple PPV’s. Thirdly, inclusion of eyes with diabetes mellitus might be a confounding factor in the present study, however, excluding these eyes did not affect the results. Finally, after the shift from 20-gauge to higher gauge PPV’s, complex eyes were mostly still operated using 20-gauge PPV, which might affect its association with the final IOP.

## Conclusion

The current study showed an increase in IOP after PPV with indication retinal detachment, while this effect was absent in the other indications for PPV. Furthermore, it showed that eyes undergoing one or more PPV’s have an almost three-fold higher risk of undergoing glaucoma surgery compared to their fellow eyes. Careful monitoring of the IOP is needed after PPV, especially in case of retinal detachment, to prevent and manage secondary glaucoma.

## Data Availability

The datasets used and/or analyzed during the current study are available from the corresponding author on reasonable request.

## Abbreviations

CI: Confidence interval
IOP: Intraocular pressure
OR: Odds ratio
PPV: Pars plana vitrectomie

## References

[1] Machemer R, Buettner H, Norton EW, Parel JM (1971). Vitrectomy: a pars plana approach. Trans Am Acad Ophthalmol Otolaryngol.

[2] Machemer R, Parel JM, Norton EW (1972). Vitrectomy: a pars plana approach. Technical improvements and further results. Trans Am Acad Ophthalmol Otolaryngol.

[3] Schwartz SG, Flynn HW (2006). Primary retinal detachment: scleral buckle or pars plana vitrectomy?. Curr Opin Ophthalmol.

[4] Mehta S, Blinder KJ, Shah GK, Grand MG (2011). Pars plana vitrectomy versus combined pars plana vitrectomy and scleral buckle for primary repair of rhegmatogenous retinal detachment. Can J Ophthalmol.

[5] Kang KT, Kim KS, Kim YC (2014). Surgical results of idiopathic and secondary epiretinal membrane. Int Ophthalmol.

[6] Parravano M, Giansanti F, Eandi CM, Yap YC, Rizzo S, Virgili G. Vitrectomy for idiopathic macular hole. Cochrane Database Syst Rev. 2015;(5):Cd009080. 10.1002/14651858.CD009080.pub2. 10.1002/14651858.CD009080.pub2 PMC6669239 25965055

[7] Oyakawa RT, Michels RG, Blase WP (1983). Vitrectomy for nondiabetic vitreous hemorrhage. Am J Ophthalmol.

[8] Early Vitrectomy for Severe Vitreous Hemorrhage in Diabetic Retinopathy: Two-Year Results of a Randomized Trial Diabetic Retinopathy Vitrectomy Study Report 2 The Diabetic Retinopathy Vitrectomy Study Research Group. Arch Ophthalmol. 1985;103(11):1644–52. 10.1001/archopht.1985.01050110038020. 2865943

[9] Delaney YM, Oyinloye A, Benjamin L (2002). Nd:YAG vitreolysis and pars plana vitrectomy: surgical treatment for vitreous floaters. Eye (Lond).

[10] Jackson TL, Nicod E, Angelis A, Grimaccia F, Prevost AT, Simpson AR, Kanavos P (2013). Pars plana vitrectomy for vitreomacular traction syndrome: a systematic review and metaanalysis of safety and efficacy. Retina.

[11] Weinberg RS, Peyman GA, Huamonte FU (1976). Elevation of intraocular pressure after pars plana vitrectomy. Albrecht Von Graefes Arch Klin Exp Ophthalmol.

[12] Han DP, Lewis H, Lambrou FH, Mieler WF, Hartz A (1989). Mechanisms of intraocular pressure elevation after pars plana vitrectomy. Ophthalmology.

[13] Chang S (2006). LXII Edward Jackson lecture: open angle glaucoma after vitrectomy. Am J Ophthalmol.

[14] Wu L, Berrocal MH, Rodriguez FJ, Maia M, Morales-Canton V, Figueroa M, Serrano M, Roca JA, Arevalo JF, Navarro R (2014). Intraocular pressure elevation after uncomplicated pars plana vitrectomy: results of the Pan American collaborative retina study group. Retina.

[15] Fujikawa M, Sawada O, Kakinoki M, Sawada T, Kawamura H, Ohji M (2014). Long-term intraocular pressure changes after vitrectomy for epiretinal membrane and macular hole. Graefes Arch Clin Exp Ophthalmol.

[16] Mi CW, Thompson JT (2015). Long-term follow-up of intraocular pressure after vitrectomy in eyes withour preexisting glaucoma. Retina.

[17] Yu AL, Brummeisl W, Schaumberger M, Kampik A, Welge-Lussen U (2010). Vitrectomy does not increase the risk of open-angle glaucoma or ocular hypertension--a 5-year follow-up. Graefes Arch Clin Exp Ophthalmol.

[18] Lalezary M, Kim SJ, Jiramongkolchai K, Recchia FM, Agarwal A, Sternberg P (2011). Long-term trends in intraocular pressure after pars plana vitrectomy. Retina.

[19] Kovacic H, Wolfs RCW, Kılıç E, Ramdas WD. Changes in intraocular pressure after intraocular eye surgery-the influence of measuring technique. Int J Ophthalmol. 2019;12(6):967-73. 10.18240/ijo.2019.06.14. eCollection 2019. 10.18240/ijo.2019.06.14 PMC6580218 31236354

[20] van der Valk R, Webers CA, Schouten JS, Zeegers MP, Hendrikse F, Prins MH (2005). Intraocular pressure-lowering effects of all commonly used glaucoma drugs: a meta-analysis of randomized clinical trials. Ophthalmology.

[21] van Koolwijk LM, Ramdas WD, Ikram MK, Jansonius NM, Pasutto F, Hysi PG, Macgregor S, Janssen SF, Hewitt AW, Viswanathan AC (2012). Common genetic determinants of intraocular pressure and primary open-angle glaucoma. PLoS Genet.

[22] Singh CN, Iezzi R, Mahmoud TH (2010). Intraocular pressure instability after 23-gauge vitrectomy. Retina.

[23] Gedde SJ (2002). Management of glaucoma after retinal detachment surgery. Curr Opin Ophthalmol.

[24] Jonas JB, Knorr HL, Rank RM, Budde WM (2001). Intraocular pressure and silicone oil endotamponade. J Glaucoma.

[25] Honavar SG, Goyal M, Majji AB, Sen PK, Naduvilath T, Dandona L (1999). Glaucoma after pars plana vitrectomy and silicone oil injection for complicated retinal detachments. Ophthalmology.

[26] Antoun J, Azar G, Jabbour E, Kourie HR, Slim E, Schakal A, Jalkh A (2016). Vitreoretinal surgery with silicone oil tamponade in primary uncomplicated rhegmatogenous retinal detachment: clinical outcomes and complications. Retina.

[27] Al-Jazzaf AM, Netland PA, Charles S (2005). Incidence and management of elevated intraocular pressure after silicone oil injection. J Glaucoma.

[28] Abrams GW, Swanson DE, Sabates WI, Goldman AI (1982). The results of sulfur hexafluoride gas in vitreous surgery. Am J Ophthalmol.

[29] Chang S, Lincoff HA, Coleman DJ, Fuchs W, Farber ME (1985). Perfluorocarbon gases in vitreous surgery. Ophthalmology.

[30] Tranos P, Asaria R, Aylward W, Sullivan P, Franks W (2004). Long term outcome of secondary glaucoma following vitreoretinal surgery. Br J Ophthalmol.

[31] Hasegawa Y, Okamoto F, Sugiura Y, Okamoto Y, Hiraoka T, Oshika T (2014). Intraocular pressure elevation after vitrectomy for various vitreoretinal disorders. Eur J Ophthalmol.

[32] Thompson JT (2011). Advantages and limitations of small gauge vitrectomy. Surv Ophthalmol.

[33] de Vries MM, Muskens RP, Renardel de Lavalette VW, Hooymans JM, Jansonius NM (2016). Glaucoma drainage device surgery after vitreoretinal surgery: incidence and risk factors. Acta Ophthalmol.

